# Vitamin D supplementation for women during pregnancy

**DOI:** 10.1590/1516-3180.20161343T2

**Published:** 2015-04-14

**Authors:** Luz Maria De-Regil, Cristina Palacios, Lia K Lombardo, Juan Pablo Peña-Rosas

## Abstract

**BACKGROUND:**

Vitamin D deficiency or insufficiency is thought to be common among pregnant women. Vitamin D supplementation during pregnancy has been suggested as an intervention to protect against adverse pregnancy outcomes.

**OBJECTIVES:**

To examine whether oral supplements with vitamin D alone or in combination with calcium or other vitamins and minerals given to women during pregnancy can safely improve maternal and neonatal outcomes.

**METHODS:**

**Search methods:**

We searched the Cochrane Pregnancy and Childbirth Group's Trials Register (23 February 2015), the International Clinical Trials Registry Platform (31 January 2015), the Networked Digital Library of Theses and Dissertations (28 January 2015) and also contacted relevant organisations (31 January 2015).

**Selection criteria:**

Randomized and quasi-randomized trials with randomization at either individual or cluster level, evaluating the effect of supplementation with vitamin D alone or in combination with other micronutrients for women during pregnancy.

**Data collection and analysis:**

Two review authors independently i) assessed the eligibility of studies against the inclusion criteria ii) extracted data from included studies, and iii) assessed the risk of bias of the included studies. Data were checked for accuracy. The quality of the evidence was assessed using the GRADE approach.

**MAIN RESULTS:**

In this updated review we included 15 trials assessing a total of 2833 women, excluded 27 trials, and 23 trials are still ongoing or unpublished. Nine trials compared the effects of vitamin D alone versus no supplementation or a placebo and six trials compared the effects of vitamin D and calcium with no supplementation. Risk of bias in the majority of trials was unclear and many studies were at high risk of bias for blinding and attrition rates.

Vitamin D alone versus no supplementation or a placebo

Data from seven trials involving 868 women consistently show that women who received vitamin D supplements alone, particularly on a daily basis, had higher 25-hydroxyvitamin D than those receiving no intervention or placebo, but this response was highly heterogeneous. Also, data from two trials involving 219 women suggest that women who received vitamin D supplements may have a lower risk of pre-eclampsia than those receiving no intervention or placebo (8.9% versus 15.5%; risk ratio (RR) 0.52; 95% CI 0.25 to 1.05, low quality). Data from two trials involving 219 women suggest a similar risk of gestational diabetes among those taking vitamin D supplements or no intervention/placebo (RR 0.43; 95% CI 0.05, 3.45, very low quality). There were no clear differences in adverse effects, with only one reported case of nephritic syndrome in the control group in one study (RR 0.17; 95% CI 0.01 to 4.06; one trial, 135 women, low quality). Given the scarcity of data for this outcome, no firm conclusions can be drawn. No other adverse effects were reported in any of the other studies.

With respect to infant outcomes, data from three trials involving 477 women suggest that vitamin D supplementation during pregnancy reduces the risk preterm birth compared to no intervention or placebo (8.9% versus 15.5%; RR 0.36; 95% CI 0.14 to 0.93, moderate quality). Data from three trials involving 493 women also suggest that women who receive vitamin D supplements during pregnancy less frequently had a baby with a birthweight below 2500 g than those receiving no intervention or placebo (RR 0.40; 95% CI 0.24 to 0.67, moderate quality).

In terms of other outcomes, there were no clear differences in caesarean section (RR 0.95; 95% CI 0.69 to 1.31; two trials; 312 women); stillbirths (RR 0.35 95% CI 0.06, 1.99; three trials, 540 women); or neonatal deaths (RR 0.27; 95% CI 0.04, 1.67; two trials, 282 women). There was some indication that vitamin D supplementation increases infant length (mean difference (MD) 0.70, 95% CI -0.02 to 1.43; four trials, 638 infants) and head circumference at birth (MD 0.43, 95% CI 0.03 to 0.83; four trials, 638 women).

Vitamin D and calcium versus no supplementation or a placebo

Women who received vitamin D with calcium had a lower risk of pre-eclampsia than those not receiving any intervention (RR 0.51; 95% CI 0.32 to 0.80; three trials; 1114 women, moderate quality), but also an increased risk of preterm birth (RR 1.57; 95% CI 1.02 to 2.43, three studies, 798 women, moderate quality). Maternal vitamin D concentration at term, gestational diabetes, adverse effects and low birthweight were not reported in any trial or reported only by one study.

AUTHORS CONCLUSIONS: New studies have provided more evidence on the effects of supplementing pregnant women with vitamin D alone or with calcium on pregnancy outcomes. Supplementing pregnant women with vitamin D in a single or continued dose increases serum 25-hydroxyvitamin D at term and may reduce the risk of pre-eclampsia, low birthweight and preterm birth. However, when vitamin D and calcium are combined, the risk of preterm birth is increased. The clinical significance of the increased serum 25-hydroxyvitamin D concentrations is still unclear. In light of this, these results need to be interpreted with caution. Data on adverse effects were lacking in all studies.

The evidence on whether vitamin D supplementation should be given as a part of routine antenatal care to all women to improve maternal and infant outcomes remains unclear. While there is some indication that vitamin D supplementation could reduce the risk of pre-eclampsia and increase length and head circumference at birth, further rigorous randomized trials are required to confirm these effects.

The full text of this review is available free-of-charge from: http://onlinelibrary.wiley.com/doi/10.1002/14651858.CD008873.pub3/epdf
